# Combining Theory-Driven Evaluation and Causal Loop Diagramming for Opening the ‘Black Box’ of an Intervention in the Health Sector: A Case of Performance-Based Financing in Western Uganda

**DOI:** 10.3390/ijerph14091007

**Published:** 2017-09-03

**Authors:** Dimitri Renmans, Nathalie Holvoet, Bart Criel

**Affiliations:** 1 Institute of Development Policy and Management (IOB), University of Antwerp, Antwerp 2000, Belgium; nathalie.holvoet@uantwerpen.be; 2 Department of Public Health, Institute of Tropical Medicine, Antwerp 2000, Belgium; bcriel@itg.be

**Keywords:** complexity, performance-based financing, LMIC, Uganda, systems thinking, theory-driven evaluation, health systems, methodology, causal loop diagramming

## Abstract

Increased attention on “complexity” in health systems evaluation has resulted in many different methodological responses. Theory-driven evaluations and systems thinking are two such responses that aim for better understanding of the mechanisms underlying given outcomes. Here, we studied the implementation of a performance-based financing intervention by the Belgian Technical Cooperation in Western Uganda to illustrate a methodological strategy of combining these two approaches. We utilized a systems dynamics tool called causal loop diagramming (CLD) to generate hypotheses feeding into a theory-driven evaluation. Semi-structured interviews were conducted with 30 health workers from two districts (Kasese and Kyenjojo) and with 16 key informants. After CLD, we identified three relevant hypotheses: “success to the successful”, “growth and underinvestment”, and “supervision conundrum”. The first hypothesis leads to increasing improvements in performance, as better performance leads to more incentives, which in turn leads to better performance. The latter two hypotheses point to potential bottlenecks. Thus, the proposed methodological strategy was a useful tool for identifying hypotheses that can inform a theory-driven evaluation. The hypotheses are represented in a comprehensible way while highlighting the underlying assumptions, and are more easily falsifiable than hypotheses identified without using CLD.

## 1. Introduction

The sustainable development goals have put universal health coverage in the midst of the world health agenda and with it health system strengthening [1]. This shift in the international policy agenda warrants a move to more health systems oriented research and evaluation. Simultaneously, the latter two domains increasingly focus on “complexity” in health systems [2]. This has resulted in many different methodological responses: the defiance [3,4] and/or adaptation [5] of existing randomized controlled trial methodologies; the application of theory of change [6]; the rising prominence of realist evaluation [7,8] and, more generally, the use of theory-driven evaluations [9,10]; and the introduction of systems thinking [11,12]. Here, we combine theory-driven evaluation, and systems thinking to tackle this complexity and open the ‘black box’ of an intervention, i.e., discover the mechanisms that lead to the outcomes.

Theory-driven evaluation emphasizes the importance of an extensive and explicit theory as a hypothesis for evaluation/research [9,10], aiming to uncover the mechanisms leading to certain outcomes and to test the intervention’s program theory. The latter is “a set of explicit or implicit assumptions by stakeholders about what action is required to solve a social, educational, or health problem and why the problem will respond to this action” [13] (p. 17). We add that insights from earlier research can further strengthen the program theory. After the creation of the program theory, the evaluation then sets out to study whether this program theory actually materialized in order to understand observed outcomes. The development of the program theory necessitates an in-depth understanding of a context characterized by complexity. However, this complexity is ignored by many program theories; instead reverting to a linear graphical visualization highly resembling the well-known “logical framework” (e.g., [14,15]) thus reproducing its shortcomings in situations of complexity [16].

Like Dr. Patricia Rogers and Bob Williams [17], we believe that a “systems thinking approach” can be a useful way to deal with this issue of complexity. More specifically, as a program theory mainly aims to uncover inter-relations between the elements of the project and the pre-existing environment, tools from the sub-field of system dynamics are especially useful [18]. Systems thinking takes a holistic approach towards interventions [11,12] as it looks at interaction effects, feedback loops, and emergence (Emergence means that the interactions between the different parts of the system lead to effects or properties at the system level that are different from those observed at the level of the constituting parts.) within the larger system, such as a health care system, instead of focusing on linear relationships between two variables. Moreover, in our approach we stress the fact that an intervention is implemented in a pre-existing system/environment/context; the intervention influences the context, not the other way around. Causal loop diagramming (CLD) is one of the tools from the systems dynamic sub-field used to analyze systems and stems from industrial management research in the 1960s [19] and organizational learning [20], but it also has some useful applications in the health sector [21,22,23,24]. CLD is a qualitative visual aid used to communicate the assumptions of a “mental model of a dynamic system” [25]. The latter is a “relatively enduring and accessible, but limited, internal conceptual representation of an external system whose structure maintains the perceived structure of that system” [26] (p. 17), i.e., a theory of the system/intervention at hand or a program theory. By visualizing these assumptions, the approach aims to better understand the behavior of the system and its agents. As the name implies, a specific focus of this tool is on discovering feedback loops in the system. Similar to the program theory within theory-driven evaluation, it relies on the inputs of relevant stakeholders, beneficiaries, key informants and the researcher’s knowledge of earlier studies. Therefore, it is very well suited to guide the definition of a program theory at the beginning of a theory-driven evaluation.

By using CLD, a well thought-out program theory can be devised. In turn, hypotheses can be generated which feed into a theory-driven evaluation, leading to a more in-depth evaluation and knowledge of the mechanisms at play.

To showcase this, we analyze the implementation of a performance-based financing (PBF) intervention of the Belgian Technical Cooperation (BTC) in the Rwenzori and West Nile regions of Western Uganda. PBF has increased in popularity as way of improving the performance of the health care system in low- and middle-income countries. In its broadest sense, PBF is a results-oriented reform package composed of financial incentives (for facilities and/or health workers based on quality measures, and the number of targeted services), and some or all of the following elements: a separation of functions, (spending) autonomy for the health facilities, strict monitoring and verification of services, community involvement, results-oriented planning, and accountability arrangements (see [27] for a discussion on the broad and narrow definitions of PBF).

Although PBF has achieved some remarkable results, especially in Rwanda [28], the overall outcomes and effects of this intervention are mixed [29,30]. Gaining insights into the mechanisms that lead to success and failure will help practitioners improve the design of PBF schemes and other health interventions. The use of a systems thinking approach was proposed several years ago, but with little follow-up [31]. This paper contributes to filling this gap. Moreover, as observed by Marchal et al. [32], multi-component interventions (like PBF) may elicit difficulties of attribution when using theory-driven evaluations, more specifically realist evaluation. We believe that the use of CLD may help to overcome this as it is specifically designed to address the issue of multi-causality. Being a reform package with several components, PBF is thus the ideal test case.

This study is part of broader research evaluating the implementation of BTC’s PBF intervention. The study received ethical approval from the institutional review boards of Makerere University School of Public Health (protocol 334, 11 September 2015), the Institute of Tropical Medicine of Antwerp, and the University Hospitals of Antwerp (B300201525175, 13 July 2015).

## 2. Materials and Methods

### 2.1. Study Setting

The study was set in Uganda, one of the latest sub-Saharan African countries planning to roll out a nationwide PBF scheme. Uganda is also one of the sub-Saharan African countries that failed to achieve the health-related Millennium Development Goals [33] and has a health care system that suffers from understaffing, low wages, poor supervision, regular drug depletion at facilities, and poor logistics management [34,35]. With a maternal mortality ratio of 343 per 100,000 live births, a healthy life expectancy at birth of 54 years, and an under-five mortality rate of 54.6 per 1000 live births, Uganda ranks 146th, 156th, and 155th out of 194 countries, respectively [36].

The health sector is comprised of three types of facilities providing modern health care: public, private not-for-profit (PNFP), and private for-profit. This study focuses on the PNFP sector, which comprises 40–50% of the health care facilities in Uganda [37,38] and consists mainly of the health facilities from the Ugandan Catholic Medical Bureau (UCMB), Ugandan Protestant Medical Bureau (UPMB), and Ugandan Muslim Medical Bureau (UMMB). Given the decentralized nature of the Ugandan health system, these medical boards have a certain level of autonomy, but the District Health Management Team remains an important oversight body. Each facility has a Health Unit Management Committee that guides and oversees the management of the facility and is composed of representatives from the community, local government, and health facility.

Due to their institutional location, PNFP facilities receive fewer public funds than the government facilities. Health workers in the PNFP sector are generally paid from the patients’ user fees, whereas health workers at government facilities receive a fixed salary from the government. Although some facilities in Kasese district have staffs that are on government payroll (see below). Since 2001, health care services are, in principle, free of charge at government facilities; by contrast user fees are still paid at PNFP facilities except for certain services, such as antenatal care and vaccinations, among others. Notwithstanding the lower budget and lower salaries in the PNFP sector, the latter is considered to be delivering higher quality services than the government facilities, mainly due to regular medicine stock outs in the latter [37,38]. Given their important role in delivering health care services to the general public (including the poor and rural areas), the Ugandan PNFP facilities are sometimes referred to as “quasi-public organizations” [39].

**Figure 1:**
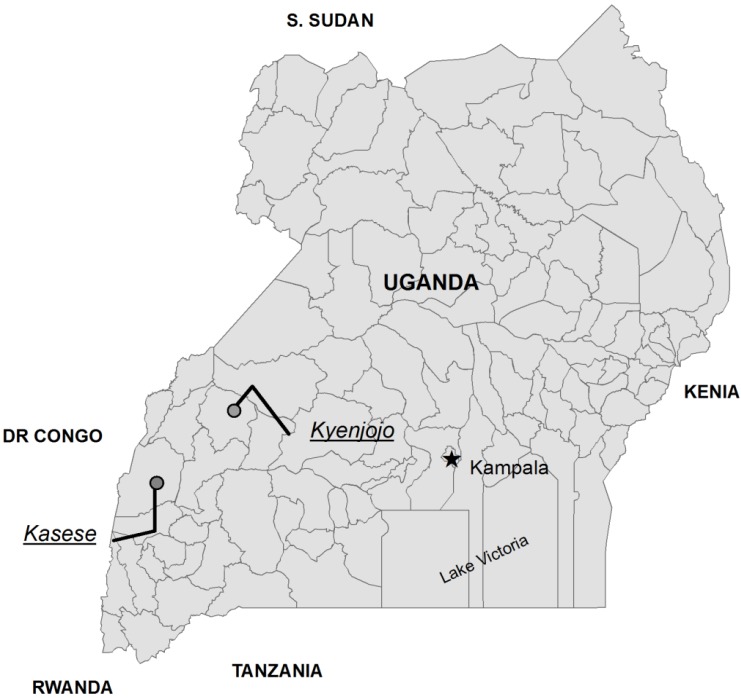
Map of Uganda and districts of interest.

Uganda’s far-fetched decentralization is noticeable in the organization of its health care sector; starting from the Village Health Teams, it goes up to Health Centers (HCs) II, III, and IV (the latter is either a hospital or an upgraded health center and the focal point of a sub-district) and the regional and national referral hospitals. This study focuses on HCs III and IV from the catholic and the protestant medical bureaus in two districts of the Rwenzori region in Western Uganda: Kasese and Kyenjojo (Figure 1). Table 1 lists the most relevant differences. Importantly, a significant part of the health workers in the Kasese district are government-seconded staff paid according to the higher government scales, whereas all Kyenjojo district health workers are paid from patients’ user fees. In addition, the number of facilities that managed to qualify for the intervention (see below) differs significantly between the two districts.

### 2.2. BTC’s PBF Intervention

The BTC intervention is restricted to HCs III and IV and regional hospitals of the PNFP sub-sector. Health facilities go through a pre-qualification assessment of the level of infrastructural quality (e.g., presence of incinerator, waste bins, etc.), with three possible outcomes: qualification for the PBF intervention, conditional qualification (facility receives a minor investment according to a performance improvement plan aimed at achieving the criteria), and re-assessment 6 months after implementation of an investment plan (receives no intervention funds). Selected health facilities are eligible to sign a PBF contract, receive a one-time investment for drugs and small equipment, and receive quarterly extra funds based on their infrastructural quality and number of qualitative services provided (i.e., their performance). A more elaborate description of the intervention according to the “descriptive framework” (A framework that indicates which elements need to be described in order to enhance comprehension and appropriately contextualize the intervention.) proposed previously [27] can be found in Supplementary Material 1 and Section 3.8.

### 2.3. Data Collection and Analysis

Ideally, program theory and CLD are based on a thorough needs assessment of the health care environment among local stakeholders [41]. Here, we performed CLD (using Vensim© PLE version 6.4 software, Ventana Systems Inc., Harvard, MA, USA) on the basis of qualitative interviews with health workers in order to visualize their “mental model” of the health system (step 1). As we claimed in the introduction that an intervention is always introduced in a pre-existing environment, this causal loop diagram serves as the basis in which we will later introduce the PBF intervention. We then performed qualitative interviews among KIs (respondents from the Ministry of Health, BTC, Medical Bureaus, and districts), asking them about how the PBF intervention will affect the system identified in step 1 (step 2). We strengthened step 2 by including insights from studies on PBF described in a recent literature review [30] (step 3). Finally, we analyzed several feedback loops that may serve as hypotheses (step 4).

The data collection among health workers (step 1) was performed by the first author in September–October 2015, before the start of the PBF intervention and before facilities knew the outcome of the pre-qualification assessment. In each of the 15 facilities visited, the first author conducted one to three semi-structured interviews with health workers present at the facility. All interviewees were informed about the general study objectives and assured that disclosed information was treated confidentially without any influence on their job or BTC’s evaluation of the facility. We emphasized that we were university researchers not affiliated with the BTC. The interviews were conducted in English, which is the language of instruction in medical schools.

The topics on which the respondents’ opinions were asked relate to our definition of PBF and include their motivation, recordkeeping, supervision, community participation, and work environment. In order to obtain a clear view of what they considered major dysfunctions in their health care environment, we interrogated them on the causes of one specific health problem, maternal mortality. This enabled us to divert from the theoretical needs of the health care system and forced the health workers to think about the real barriers to adequate health care. We chose the example of maternal mortality because maternal services are often targeted by PBF schemes [42,43], which also holds for this PBF intervention, and because it is a health issue known for requiring a system-wide approach. Afterwards, the interviews were transcribed verbatim and coded using the open-coding technique in QSR Nvivo© 10 software (QSR International Pty Ltd., Doncaster, Australia).

A total of 30 semi-structured interviews were performed with predominantly female health workers from the different health facilities (Table 2). Though there was a relatively good balance between the different cadres, half of the respondents were nurses or nursing officers. This proportion largely matches the characteristics of the reference population. We also have no reason to assume that the health workers from the night shift (not included in the sample) would differ significantly from those on the day shift, as a rotating system is in place.

The semi-structured interviews with the KIs (step 2) were conducted by the first author during March 2017. Questions concerned the details of the intervention and how the respondents expected the intervention to have an impact on the health care outcomes. Respondents were chosen purposefully in order to interview those most closely involved in designing and implementing the intervention. We performed 16 interviews with KIs, including high-level officials within the MoH, BTC, and medical bureaus, as well as key stakeholders from the district level (Table 3).

### 2.4. Causal Loop Analysis

In CLD, variables are presented and causally linked to each other using arrows. Each arrow has a direction and polarity: “+” means that a change in the first variable in a certain direction causes a change in the second variable in the same direction compared to the situation without the change in the first variable, whereas “−“ means that the change occurs in the opposite direction. Some causal effects take time to manifest, and this delay is represented by a double line on the arrow (//). The causal loop diagrams in this paper are simplified to an extent (e.g., incentives and basic salary are combined in one variable “salary”) to facilitate interpretation. Causal loop analysis entails examination of the diagram in order to find feedback loops that are either positive (reinforcing) or negative (balancing), as shown by a clockwise or counterclockwise arrow with an “R” or “B” in the middle, respectively.

### 2.5. Limitations

This study has a number of limitations. First, even though we strongly emphasized that we were independent researchers, health workers may have had the impression that we were representing the BTC. This may have resulted in socially desirable answers. Second, we focused on the health workers’ perceptions to identify the context and the perception of higher-ups to identify the effect of the PBF intervention, which may be different from the reality perceived by other stakeholders (e.g., the community), or even the actual reality (e.g., workload [44]). However, perceptions are often the drivers of behavior, making them important. Third, we had to trade-off between “breadth” and “depth”. As we decided to discuss the entire set of PBF elements, we went for the first option. Fourth, we mainly focused on information found in studies on other PBF interventions, the analysis can and should be extended to include other programs with similar mechanisms as well. Finally, CLD is focused on feedback loops, but these are not the only points of interest and other types of relationships (e.g., linear, logarithmic, parabolic) deserve equal attention.

## 3. Results

### 3.1. CLD Set-Up

**Figure 2:**
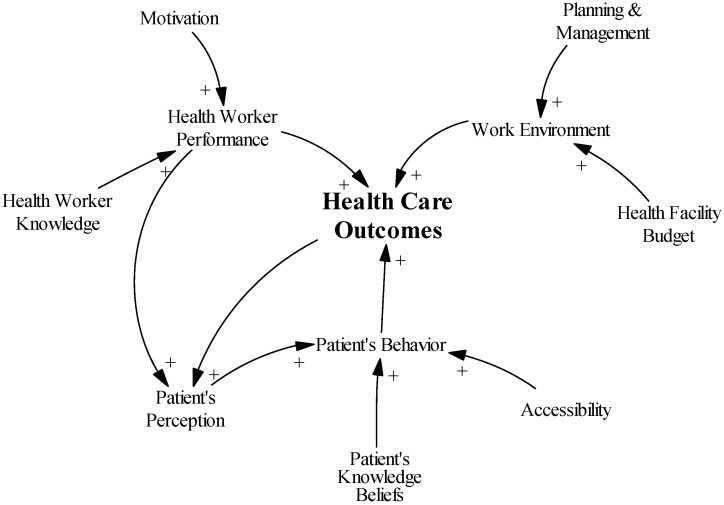
The set-up of the causal loop diagram.

The main objective and outcome variable is improved health care outcomes. Therefore, it is in the center of the diagram (Figure 2). For the sake of this exercise, we assumed that the main contributors to better health care outcomes are health worker performance, which comprises (yet not represented in the diagram) the health workers’ attitudes toward the patients, appropriateness of the care provided, and correct implementation of the treatment plan. The second and third contributors are the work environment, which entails the available equipment, infrastructure, and drugs, and patient behavior, which includes health care-seeking behavior and therapy loyalty.

Taking another step back, the performance of health workers is influenced by their motivation and knowledge. The work environment is co-determined by the health facility budget and the capacity of management to plan and implement. Patient behavior is a result of patients’ positive or negative perceptions of the facility, which are influenced by the health care outcomes and health worker performance, patient knowledge and beliefs, and the accessibility of the facility, including financial, geographic, culture, and social accessibility.

### 3.2. Motivation

Our interviews underscored that the health workers’ motivation is influenced by many sources. Many of the health workers were intrinsically motivated by the tasks they perform; however, the recognition they receive from the community is also an important driver of motivation. A bad work environment was seen as an important de-motivator. Similarly, almost all of the health workers felt that they do not earn enough (salary). There was no difference between staff on government payroll and those paid from user fees, despite the difference in salary.

“I wouldn’t say we are extremely happy with what we are paid.”

*(Resp. 2 Clinical Officer)*

“I feel good when I’m putting on my white… (LAUGHS) and the way I am serving patients and the way how the community is appreciating…”

*(Resp. 21 Nurse)*

Financial incentives based on performance were not well understood by the health workers, but among the 10 who understood the concept, 7 thought that they would help motivate health workers.

“Those incentives are good. They are good, but we do not have them […] They are good because they improve on one’s motivation.”

*(Resp. 14 Nurse)*

Benefits, other than salary, that make the government sector more attractive are higher job security, pension, and study leave opportunities.

“Of course government sector is the best because of their payment. They can also give you a study leave.”

*(Resp. 21 Nurse)*

### 3.3. Recordkeeping

Respondents listed a diversity of reasons for the importance of recordkeeping. Some highlighted its utility for planning at the health facility level, others thought it improves patient care, that it is useful for surveillance, informational purposes, and accountability, and that it can also trigger motivation.

“It helps us in planning. It helps us in making decisions—like we are able to know that we have such a number of patients […] who will need such an amount of treatment or drugs.”

*(Resp. 6 Nursing Officer)*

“We have to keep it to show that we are working.”

*(Resp. 20 Nurse)*

These different uses for recordkeeping can be categorized under the two generic functions of monitoring/recordkeeping systems: the feedback/learning function (using it for planning at the facility level and patient care) and the accountability function (reporting to higher authorities or the community) [45]. The feedback/learning function was mentioned by 24 of the 30 respondents, whereas 17 respondents highlighted the accountability function. However, only three of these respondents explicitly referred to the community as the target audience, though all of them referred to upward accountability.

Although the majority of the respondents stated that the recordkeeping is easy and does not impact the workload much, probably due to the low patient flow at the visited facilities, 10 out of 11 respondents highlighted that computerization of the recordkeeping may simplify the work, especially if patient numbers increase.

“[O]nly sometimes the challenging part of it is when you are overloaded with work […] but in general the data, the way we are collecting it, doesn't need much effort or time.”

*(Resp. 11 Midwife)*

### 3.4. Supervision

Almost all of the respondents (24 out of 28) spontaneously highlighted the importance of qualitative supervision for health worker knowledge and performance. The majority of the respondents preferred formative and personal supervision over a cursory checklist evaluation of the data in the registers. One respondent argued that the latter may be caused by too high of a workload for the supervisors. Although respondents were satisfied with the supervision by the district health management team, they felt that it should be more frequent. Yet, they were aware that this requires sufficient resources.

“Supervision helps us to improve on the quality of work.”

*(Resp. 11 Midwife)*

“Others […] They just come and give you a talk and they go [without] even checking what has gone wrong and correct you.”

*(Resp. 19 Nursing Officer)*

Only 10% of the interviewees spontaneously mentioned motivation as an important outcome of the supervision they currently receive. Such motivation comes mainly from close interactions with the supervisor and the feedback received, which emphasizes the importance of close and formative supervision.

“It is so encouraging like when I am doing work and they come in to supervise me and tell me I'm doing a good work.”

*(Resp. 10 Clinical Officer)*

### 3.5. Community Participation

The health workers who were interviewed highlighted three not mutually exclusive roles for the community within the health unit management committee: an informational role, which implies reporting of the concerns, needs, and wishes of the community to the management committee and health workers with the aim to improve the appropriateness and quality of the health services provided; a dissemination role, disseminating information and helping with sensitization within the community, but as mentioned above the information does not necessarily relate to the facility’s performance; and a decision making role. In general, the first two roles are welcomed by the health workers, whereas the capacity of the community to take on a decision-making role is doubted (e.g., the construction of a new building). The majority of the respondents felt that the main decision-making power should lie with the health workers who, according to the health workers themselves, have sufficient technical capacity and knowledge, unlike the community.

“No, they should have less [decision power] simply because they are not the technical persons.”

*(Resp. 14 Nurse)*

### 3.6. Working Environment

While visiting the facilities, we found that very few patients (often <10 daily) attend them. Among the most likely explanations is the competition from government health facilities, which provide free health care. Moreover, given the rural context, patients have to travel greater distances to come to the facility and have fewer financial resources compared to more urban areas. This low number of patients is not only problematic for the health facility budget, and consequently the work environment, but also for the health workers’ motivation to be effectively present at the health facility.

“[T]oday I have seen only two people […], so if there are supposed to be two health workers on duty, you cannot both come to see two people.”

*(Resp. 5 Clinical Officer)*

However, in facilities with several mothers coming in for antenatal care and many HIV patients coming in for treatment, both of which are free services (50 patients for two afternoons per week), there are complaints about the heavy workload due to the low staffing level (see also Zakumumpa et al. [46]). This is especially evident in the maternity ward, where only one midwife is often responsible for all maternal health services. However, vacancy levels differ greatly between facilities, and even departments (e.g., maternity and out-patient departments) [47]. Times of high workload have consequences on the health workers’ performance and their motivation, and puts pressure on the work environment (equipment, space, drugs, etc.).

“I will give an example of the maternity ward. You find someone is attending to a mother in labor, soon you find that the one at [the delivery] is the one who is supposed to attend to those who have already delivered. So the work that should be done by four people is being done by one person.”

*(Resp. 2 Clinical Officer)*

Health workers also highlighted inadequate infrastructure (13 respondents) and equipment (16 respondents), including the lack of sufficient qualitative staff quarters, patient wards, and appropriate equipment and drugs, which forces them to improvise or send the patients to private medical stores.

“[I]f those sets are not there we improvise with the […] razor blade.”

*(Resp. 17 Nurse)*

### 3.7. Maternal Mortality

Demand-side barriers are the most referenced causes of maternal mortality. More than half of the respondents mentioned poverty or the lack of financial resources, as this leads to bad health conditions (malnutrition, sickness), increasing the risk of complications during birth. Moreover, the cost of transportation, the facility user fee, and the consumables they have to buy, pushes poor women to deliver with a traditional birth attendant rather than at a facility, which is considered the safer option.

“[T]he crises of money […] they get anemia, malnutrition which can complicate labor, they can end up dying.”

*(Resp. 13 Nurse)*

Half of the health workers blame the ignorance of mothers. Traditional beliefs, a lack of knowledge, and stubbornness are considered reasons why mothers deliver in the villages with the help of traditional birth attendants instead of at a health facility, or come to the facility when it is already too late.

“They have their traditional beliefs […], they are those with a rigid mind even though you teach, they will become stubborn.”

*(Resp. 19 Nursing Officer)*

In addition to these demand-side barriers, the health workers also highlighted supply-side factors. An important issue highlighted by 16 respondents was the poor geographic accessibility of the health facilities. A problem compounded by the lack of ambulances at the facility level and the financial barriers to transport.

“[P]eople come from mountainous areas and you find the transportation itself being bad, and ambulance services still low.”

*(Resp. 11 Midwife)*

The causes of maternal mortality pointed out thus far are based on the assumption that not delivering at the facility is detrimental to the outcome of the delivery. However, even when pregnant women manage to get to the health facilities, problems may still arise due to other obstacles, such as lack of equipment, infrastructure, and qualified health workers (highlighted by 14 respondents). Sub-standard quality services caused by poor health worker attitudes are very likely an important contributing factor that tends to be underestimated by most health workers (only six respondents mentioned it).

“Also health workers, they are there who are quarrelsome to mothers. And you find that the mothers fear to express the way she is feeling.”

*(Resp. 4 Midwife)*

**Figure 3:**
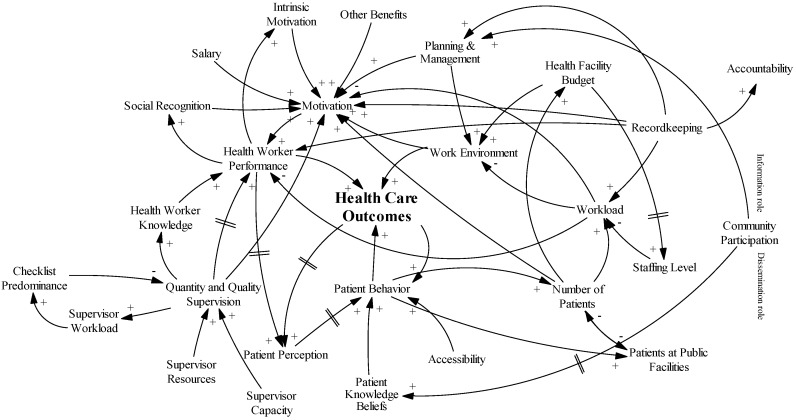
Causal loop diagram as perceived by the health workers.

Figure 3 shows the causal loop diagram obtained after assessing the needs of the health care environment as perceived by the health workers. As not all variables have an immediate effect, we included delay marks where needed. Importantly, the health facility budget only has a positive effect on the work environment and number of health workers from a certain level onward because an important part of the budget is reserved to cover recurrent costs.

### 3.8. Introducing the PBF Intervention

**Figure 4:**
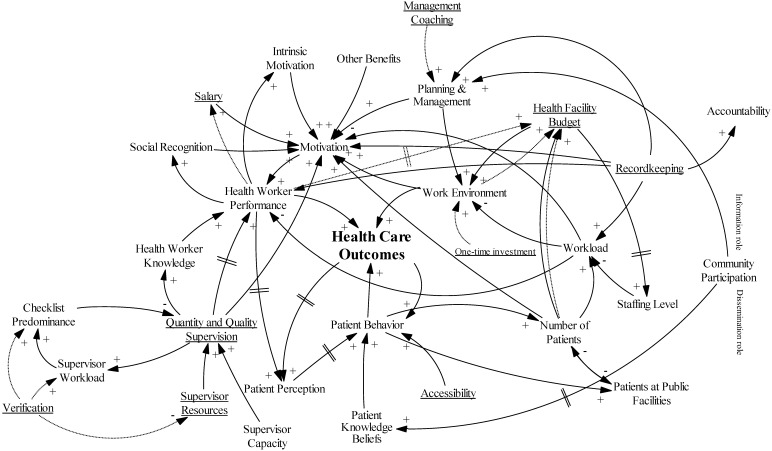
Causal loop diagram with the introduction of PBF.

As discussed elsewhere [27,48], PBF is a reform package that intervenes at different points of the health care system. In this section, we integrate BTC’s PBF intervention into our causal loop diagram (Figure 4) using input from both the KI interviews and existing knowledge from the literature (steps 2 and 3). The dotted lines and underlined text in Figure 4 represent the direct influence of the PBF intervention. As already stated, a thorough description of the intervention can be found in Supplementary file 1.

Central to every PBF intervention is that it creates a link between pre-defined measures and the budget/salary. In our specific case, the PBF bonus to the health facility budget is based on the quality of the work environment, the performance of the health worker, and the number of patients treated. Twenty-five percent of the PBF bonus can be used for health worker incentives (salary) based on individual performance.

Another important aspect of this PBF intervention is that the funds are seen as a subsidization of the user fees, which are subsequently reduced. This leads to increased financial accessibility and more patients coming to the facility (KIs 1, 4, 7–9, 11–15). As discussed in the description of the intervention, facilities that are qualified get a one-time investment to finance medicines and basic equipment, strengthening the work environment (KIs 5, 6, 12, 15, 16).

PBF also increases the importance of recordkeeping. Notwithstanding the intervention’s use of the existing registers, we can still expect a small increase in the workload [49]. The intervention also encourages the use of the records for facility planning and management, which is strengthened through coaching and training and introduction of the “business plan” (KIs 5, 6, 9, 11, 13). Not only does the facility management receive support, the intervention also incentivizes more supervision of the health workers by the district health management team, leading to more resources (KIs 13 and 15). The intervention introduces extra visits to verify the indicators, which impacts the workload and resources needed (KI 12). The verification also introduces a tendency for supervision along the lines of a checklist based on the national guidelines (KI 13).

The intervention does not give a specific role to the community other than what already existed, namely “co-managing” the facility in the health unit management committee (KIs 1, 2, 4, 5, 11).

### 3.9. Causal Loop Analysis

Depicting these different causal linkages in one diagram results in a very complex and difficult to interpret tangle of arrows (see Figure 4). However, causal loop analysis is about discovering the reinforcing and balancing feedback loops within a system, and using the appropriate command in the software easily lifts out these different loops. In this section, we will discuss three loops that can be used as a hypothesis for theory-driven evaluation. To clarify the loops, we have simplified the general diagram by focusing on the specific loop, deleting some variables and arrows, and bringing some variables together in one box.

#### 3.9.1. Hypothesis 1: “Success to the Successful”

**Figure 5:**
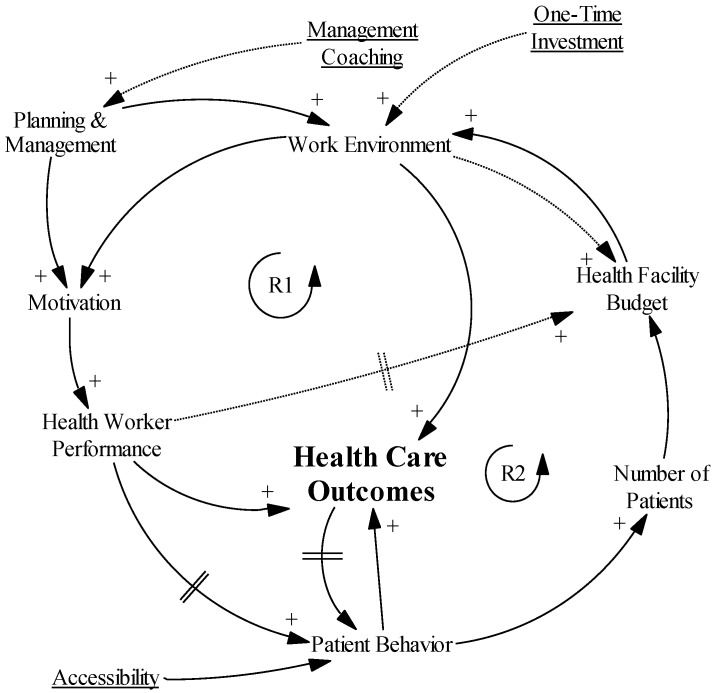
Causal loop diagram of “success to the successful” hypothesis.

Figure 5 depicts two reinforcing loops (R1 and R2) that together resemble one of the eight archetypes of systems thinking, the “success to the successful” systems archetype [50]. It starts with the one-time investment improving the work environment (basic equipment and stock of medicine), which positively influences the motivation and performance of the health workers [49,51,52]. This leads to improvements in the health facility budget (R1), health care outcomes, and patient behavior (number of patients) (R2) [53,54], with some delay in the latter. However, due to the improved financial accessibility, the number of patients improves immediately (KIs 1, 4, 7–9, 11–15) [54]. The improved work environment and health worker performance combined with the increased number of patients increases the health facility budget, which can trigger further improvements in the work environment (KIs 1, 4, 5, 7, 8, 11–14), closing the reinforcing feedback loop. An important influencing factor of this loop is the ability of the management team to plan well and to use the newly received funds in an efficient and effective way (KI 2) [51,55,56].

#### 3.9.2. Hypothesis 2: “Growth and Underinvestment”

**Figure 6:**
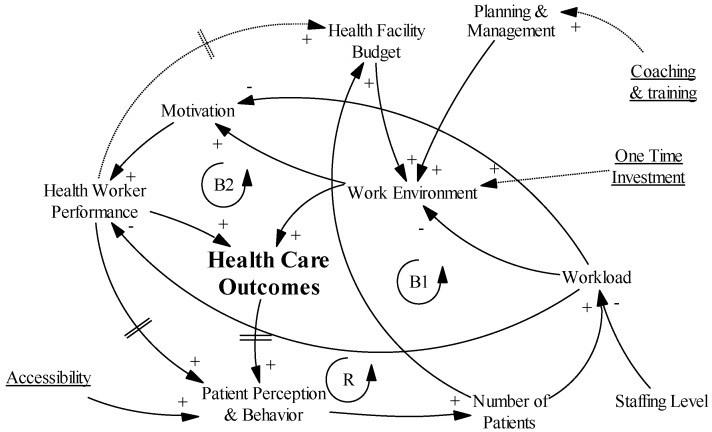
Causal loop diagram of “growth and underinvestment” hypothesis.

Figure 6 shows that this “success to successful” loop (R) may be hampered by two balancing feedback loops; the increased number of patients also increases the workload, which puts both the work environment (B1) and motivation (B2) under stress (KIs 11, 12, 14, 15) [52]. This combination of loops corresponds to the “growth and underinvestment” archetype in systems thinking [50].

More specifically, the increased workload may reduce the quality of the work environment (B1) (KI 11). Though smaller investments can be covered by the increased resources, they may not be sufficient to tackle large-scale infrastructural improvements, such as a bigger ward (KIs 11, 14, 15). (Funds coming from the PBF project are not even allowed to be invested in new infrastructure). With the wards becoming too crowded, the quality decreases, leading to fewer funds from the PBF. Fewer funds may jeopardize the investments needed in the work environment, leading to lower health care outcomes. However, the effect on patients’ perceptions will be delayed and a high number of patients remain, but the facility will only receive the reduced user fees and not the PBF incentives, as the quality will not be high enough (KI 11). In the worst-case scenario, the quality will decrease even more, eventually keeping the patients away and reducing the workload again, making it possible for the facility to perform again, eventually leading to an oscillating effect. Another possible outcome is that the facilities will increase the user fees again.

The increased workload also has a negative effect on health worker motivation and, consequently, their performance (B2) (KI 14) [49,57]. If the health facilities do not have the opportunity to hire/receive more staff, the increased workload will serve as an important bottleneck.

#### 3.9.3. Hypothesis 3: “the Supervision Conundrum”

**Figure 7:**
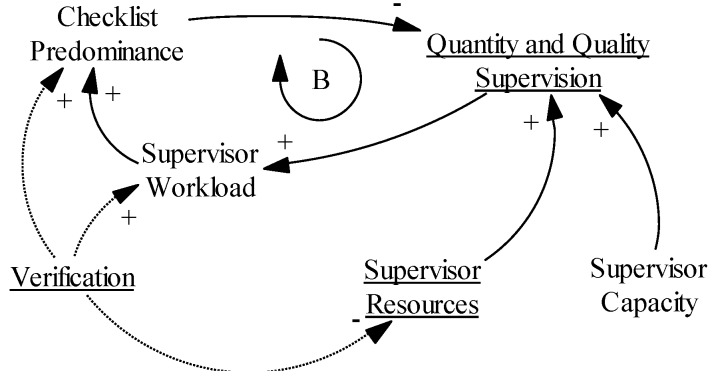
Causal loop diagram of “supervision conundrum” hypothesis.

Figure 7 focuses on the supervision part of the causal loop diagram. Again, we can discern a balancing loop (B), which is fueled in this case by external input from the intervention. Adding the verification task impacts the workload of the supervisors and the resources available, and fuels the checklist rationale (KIs 12, 13, 15) [58,59,60,61]. This may lead to less focus on the formative part of supervision [61,62]. Adding resources and reducing the workload may give more room for the formative part of the supervision (quality supervision), whereas strengthening the capacity of the verifiers will make them more capable of differentiating between their role as verifier and formative supervisor.

## 4. Discussion

Theory-driven evaluations start with stating the intervention’s program theory, which is often inferred from the intervention documents or interviews with the implementers. However, the program theory is not always written down in documents, or even present in the minds of the implementers [41] and, even if it is, it is not always based on a thorough analysis of the existing local system or a deep understanding of the intervention. For example, many of the KIs mainly focused on the incentive part of the intervention and not on the introduction of the business plan, while the issues of increased workload and strained work environment were hardly touched upon. The proposed combination of theory-driven evaluation and CLD had great potential in helping evaluators and researchers identify possible program theories based on existing knowledge from the literature and insights from local stakeholders and KIs. It did this by clarifying links, assumptions, feedback loops, etc.

When an evaluator endorses the complexity paradigm and theory-driven evaluation framework, we therefore advise the use of CLD to identify and visualize the intervention’s program theories. These program theories can then serve as the basis for a theory-driven evaluation. Our case study of a PBF intervention in Uganda showed the potential of this methodological strategy, by pointing out possible bottlenecks but also roads to success. Importantly, our CLD does not start with mapping out the mechanisms of the intervention but with the pre-existing environment after which the intervention is being introduced in this environment. The observed added value of this strategy is that program theories and related hypotheses are firmly based on existing literature, the perceptions and ideas of the local stakeholders and KIs, and the local environment/system/context. The explicitness of each of the steps in the loops facilitates tracking and evaluation of the hypotheses, which makes them easily falsifiable and helps explain why expected effects materialize or not. Critiques of these hypotheses are also easier to formulate, as other researchers can point out flaws and adapt the causal loop diagram. As CLD makes it possible to focus on specific steps, it will help prioritize the debate and research. Finally, the approach will help distinguish between what systems thinking calls “emergence” (1 + 1 = 3) and a mere sequence of effects leading to a larger effect (1 + 1 = 2).

Our causal loop analysis led to three hypotheses. The “success to the successful” hypothesis points to the potential of the PBF scheme to attract more patients to the facilities due to improved financial accessibility and improved work environment. Sufficient PBF funds compensate for the lower user fees, leading to improvements in the equipment and medicine stock, as seen in a Nigerian PBF scheme [49,51].

The “growth and underinvestment” hypothesis somewhat tempers the euphoria of the first hypothesis, as it focuses on the stress the increased workload puts on health worker motivation and the work environment from a specific tipping point onwards, as observed in other PBF schemes [49,57]. Therefore, the performance is expected to improve until a workload limit is reached, but it will not be able to improve further unless additional investments are made.

Finally, the “supervision conundrum” hypothesis raises the question of the ability of the supervisors to combine the additional verification role with their formative supervisor role without any loss of quality in the formative supervision. This may be due to a lack of time and resources, or due to the introduction of a new control-oriented checklist logic that may jeopardize more formative supervision. In a PBF scheme in Uganda, the extra verification role increased the workload significantly [58], and in Benin the increased workload interfered with “one-on-one coaching and comprehensive feedback to providers” [61]. Janssen and colleagues found a lack of “focus on the learning process” in Rwanda [62]. While a study in Tanzania presented a mixed picture with supervision focusing initially on data collection, but soon evolving into a more proactive problem-solving kind of supervision [63].

Though our study is limited in terms of scale and scope, the empirical findings used to build the causal loop diagram mirror those of other studies. In many other settings, health workers have been shown to perceive their salary as insufficient and strongly disapprove untimely payments [55,64,65,66,67]. Interestingly, higher level Ugandan stakeholders (e.g., Ministry of Health, donors, religious medical board) acknowledge that investments in human resources for health should be prioritized [68] while also recognizing the importance of non-financial incentives [66,69,70,71,72].

Even though our respondents highlighted the importance of the learning function of recordkeeping, other studies have found that records are often neglected and not correctly used as a tool to improve quality [73,74]. Thus, our respondents may adhere to the principle in words, but not in practice. This is somewhat confirmed by our interviews; respondents highlighted that retrieving the history of patients is useful but they could not recall the last time that they did so. Pirkle, Dumont and Zunzunegui [73] highlighted the importance of innovative technologies (e.g., ICT) in improving the use of data in care processes.

With regard to supervision, the preference for a more formative form of supervision instead of checklist-driven verification was also pointed out by Hernandez et al. [75]. Yet, in line with the study by Bosch-Capblanch and Garner [76], we conclude that the latter is still too often the reality.

The reluctance of the health workers towards “too much interference from the community” was also observed in Kenia and Benin, though the informational role was very much appreciated and perceived to enhance motivation [70]. Drawing upon health workers’ perceptions, the same study [70] also identified better equipment as main factor boosting performance, something that was also underscored by our interviewees. Our findings also confirm those of Rujumba et al. [77] in Eastern Uganda; their study pointed out that the perceived high workload is considered problematic. Yet, as Maestad, Torsvik and Aakvik [44] found, workload is a subjective concept and sometimes exaggerated by the health workers. Importantly, a Nigerian study found that PBF improved the working environment and, consequently, the morale of the health workers while simultaneously increasing the number of patients, the time needed for recordkeeping, and the workload [49].

Finally, the main barriers to the use of quality health care services, specifically maternal health care, highlighted by our respondents echoed those found by studies in Nigeria [78], Malawi [79], and Vietnam [80].

## 5. Conclusions

This paper examined the use of a methodological strategy that uses CLD as a way to identify relevant hypotheses to be used in a theory-driven evaluation of a PBF intervention in Western Uganda. Importantly, the CLD refrains from a specific intervention-focus and starts with mapping out the pre-existing environment after which the intervention is being introduced in it. This introduction into the diagram is based on insights from key informants and knowledge from the literature. We feel that, being a reform package affecting multiple aspects of the health system, PBF and the evaluation of PBF can strongly benefit from this methodology.

We identified three important hypotheses: “success to the successful”, “growth and underinvestment”, and the “supervision conundrum”. The first hypothesis means that the initial investment in the facility will lead to an improvement of the work environment and consequently of the quality score of the facility, which will lead to more funds and an even better work environment. The second hypothesis relates to the fact that an increased number of patients puts extra pressure on the work environment (e.g., the available space) and the health workers (increased workload) which may bring down the quality if not compensated with new investments. The final hypothesis highlights the danger of adding an extra verification task to the job description of the supervisors. This may crowd out the support supervision, due to an increase of the workload, less resources and the introduction of a checklist logic. These hypotheses are limited by the available information and the views of the researcher, KIs, and local stakeholders. Yet, using CLD renders the hypotheses more understandable and easier to trace during a theory-driven evaluation.

## Figures and Tables

**Table 1:** Differences between the two districts.

Kasese District	Kyenjojo District
694,992 residents 17.4% urban population Mountainous	422,204 residents 15.4% urban population More flat
Some government seconded staff	Staff paid by user fees
UCMB facilities: 3, UPMB facilities: 9	UCMB facilities: 5, UPMB facilities: 0
Facilities qualified *: 5/12 Facilities qualified w/conditions: 1/12 Facilities not qualified: 6/12	Facilities qualified: 1/5 Facilities qualified w/conditions: 4/5 Facilities not qualified: 0/5

* Qualification according to a pre-intervention assessment of infrastructural quality; sources: Uganda Bureau of Statistics (2016) [40], own observations, personal communications.

**Table 2:** Characteristics of health worker respondents (*n* = 30).

**Sex, *n* (%)**		**Ref. (%) ^#^**
Male	13 (43)	36
Female	17 (57)	65
**Cadre, n (%)**		
Clinical Officer	7 (23)	16
Nursing Officer	6 (20)	20
Nurse	10 (33)	38
Midwife	5 (17)	24
Records Assistant	2 (7)	n/a
**Facility**	**Respondents, n**	
Kyenjojo District	10	
Kyakatara *	2	
Kyembogo *	3	
Mabira *	1	
Rwibale *	2	
St.-Adolf *	2	
Kasese District	20	
Buhaghura	2	
Kanamba	2	
Kasanga *	1	
Kitabu *	3	
Kinyamaseke	0	
Kyanya	0	
Kyarhumba *	2	
Maliba	1	
Musyenene	1	
Nyabugando	3	
Rwesande	3	
St.-Paul	2	

* Affiliated with the UCMB, others with the UPMB; **^#^** Characteristics of the sample used in the quantitative survey (not discussed here), which includes all staff present at the moment of the survey.

**Table 3:** Overview of key informants interviewed.

BTC	4
Ministry of Health	4
Medical bureaus (UCMB & UPMB)	6
District Health Office	2
**TOTAL**	**16**

## Supplementary Materials

The following are available online at www.mdpi.com/1660-4601/14/9/1007/s1, Supplementary Materials 1: Descriptive framework of the project.
