# Supplementary material for: Combining Theory-Driven Evaluation and Causal Loop Diagramming for Opening the ‘Black Box’ of an Intervention in the Health Sector: A Case of Performance-Based Financing in Western Uganda
Source: Int J Environ Res Public Health. 2017 Sep 3;14(9):1007. doi: 10.3390/ijerph14091007 (PMC5615544; doi:10.3390/ijerph14091007)
Supplement: Supplementary file 1 [file ijerph-14-01007-s001.pdf]

Supplementary Material Table S1. Descriptive framework of the project.

Context	PBF Elements	
Health workers do not receive specific financial incentives. Salaries are perceived as being too low. A difference in salary exists between health workers paid by the government and those paid by user fees from the facility.<br>The PNFP facilities receive a small grant from the government with fixed budget lines (2–4 million UGX per quarter). NGOs often add resources to this budget either in kind (medicine, equipment) or by funding specific activities (meetings, outreaches). These funds can go up to 8 million UGX per quarter (Baylor Uganda), but are often restricted to one department (e.g., ART clinic). User fees complete the budget.<br>Non-financial incentives exist in awards given at facilities by NGOs for good performance in a certain area (e.g., drug store management).	Financial incentives	- Three kinds of incentives:<br>(1) Every interested facility needs to go through a qualification process in which they are scored based on structural measures. “Score > 85%” = the facility is qualified and receives a first incentive (money for equipment and medicines). “65% > score < 85%” = facilities receive a small grant in order to attain 85%. “Score < 65%” = not qualified, no financial support.<br>(2) Quality bonus = star rating (1 * to 5 *) for each facility based on a structural quality score, each star corresponds to a fixed lump sum grant depending on the administrative level of the health center. (HC III = 1 million UGX per star rating; HC IV = 2 million UGX per star rating).<br>(3) Quantity bonus = Fixed incentive for each service that was performed and recorded according to the pre-defined quality standards of the MoH.<br>- Depending on the scores, the amount received per quarter can go up to 20 million UGX for HC III and 40 million UGX for HC IV.<br>- Incentives are paid to the facility, 25% of the funds recceived can be used for performance-based incentives for the health workers.<br>- Quarterly paid after a reporting and verification process of about 6 weeks.<br>- Incentives are paid on a bank account of the facility, yet the facility needs the authorization of the district accounting officer to access it.
Every facility receives monthly and yearly targets for different services from the MoH. In most cases these are hung out in the different departments or in the waiting room. Also, each facility has a Quality Improvement team, which also puts forward working points. However, these were not always very active before the project. NGOs also often have targets in specific areas (e.g., drug store management).	Service and quality measures	- The pre-qualification measures correspond to infrastructure, equipment and human resources requirements.<br>- The quarterly qualification tool contains cross cutting issues of quality of service like hygiene and working environment, availability of medicines and consumables, staffing levels, use of guidelines and other management tools, implementation of performance improvement strategies, etc.<br>- Quality indicators and measures were drawn from the national guidelines, hence are per definition owned by the country.<br>- The timeframe of the measures is 3 months.
Facilities are supposed to report in the HMIS books of the MoH and make a monthly report which is send to the district. Most HC III do not have computers for the records office nor a specific records assistant. The HC IV and some HC III do have a computer and a records assistant. However, the hard copies are still needed which creates double work.	Monitoring and verification system	- The facilities first perform a self-assessment of their own performance, after which they are being visited by the extended-district health management team (E-DHMT = DHMT + representative from the concurrent medical bureau) for verification.<br>- Verifiers are not necessarily medical doctors.<br>- Performed each quarter.<br>- Quantity measures are drawn from the registers.<br>- No extra costs are envisaged, yet the district receives incentives that can be used to cover the costs of the visits (e.g., car fuel).

Table S1. *Cont*.

The MoH is both purchaser and provider. Oversight is mainly done by the district health office, the sub-district and the local government. PNFP facilities are also answerable to their respective religious medical bureau which is the owner of the PNFP facilities.	Split of functions	- The split of functions is limited. The MoH/MoFPED is envisaged to be the purchaser although in the project the BTC is the natural fund holder/purchaser. The provision is being done by the facilities under the aegis of the medical bureaus. The health coordinators of the latter together with the DHMT are responsible for the verification. To avoid collision, the coordinators of the medical bureaus will not be present during the verification visits of their own facilities.
PNFP facilities have more autonomy than public facilities. Whereas services at public facilities are free of charge and these facilities thus receive all their funds from the government through fixed line budgets, the PNFPs receive only a small part of their budget from the government with fixed budget lines. They have full autonomy on the use of the received user fees and free donations. Funds from NGOs are mostly earmarked, not giving any autonomy to the facility (e.g., Baylor Uganda).	Autonomy	- Facilities cannot use the budget for infrastructural investments or salary top ups with no link to performance.<br>- Facilities have to use the funds as fixed in the business plan, which they compose themselves with support from the district.<br>- The qualification bonus can only be used for medicines and small equipment in order to bridge the first quarter in which no funds are received yet. Only a fixed part of the received funds (25%) can be used for performance-based incentives for the health workers.<br>- In order to retrieve money from their bank account facilities have to justify to and get permission from the district accountancy officer.
Information board often have statistics on the performance of the health facility, however, these are not always accurate or up-to-date. Accountability is mainly towards the health unit management committee (HUMC) and the district.	Accountability arrangements	- Facilities are obliged to communicate their results, exact prices and the money received to the community via their information boards.<br>- Performance reports are send to the district which aggregates the data and sends it through to the national level.
The community is involved through their representatives in the HUMC. The UCMB has its own patient satisfaction surveys.	Community involvement	- Community involvement is very limited. No new tasks were assigned to the community. The community is represented in the HUMC via their local leaders. The HUMC has to underwrite the business plan. No incentives are given to the community leaders, nor are their incentives based on the participation of the community.<br>- Patient-satisfaction surveys will be performed but incentives are not based on them, they are purely informative.
The activity of the HUMC differed strongly across facilities. However, no explicit business or strategic plan was used in most of the facilities.	Planning arrangements	- The business plan is meant to set out how the facility aims to reach their goals using the expected PBF funds. It is focused on the PBF indicators and the three month timeframe.<br>- Misappropriation or misuse of funds is being sanctioned<br>- Business plan is approved by the HUMC and the district health office

Table S1. *Cont*.

All the facilities of the medical bureaus go through a light accreditation process.<br>Workshops and meetings exist with mainly the in-charges attending them.<br>The districts are seen to be lacking the capacities to really support the facilities in there functioning.<br>Health workers strongly appreciate the supervision received and often stated that they would like to welcome the supervisors more often.<br>The user fees at the PNFP facilities were seen as important barriers to access to care. Since services at public facilities are free, many patients divert to them instead of the PNFP facilities. This lead to a very low number of patients in the PNFP facilities.	Ancillary components	- Certification: every interested facility needs to go through a pre-qualification process in which it performs a self-assessment after which this assessment is being verified. Facilities with a score of 85% and above are allowed in the project. This is like a certification process.<br>- The in-charges of the facilities receive workshops on the specificities of the project which they have to communicate to their staff.<br>- The district PBF focal persons have quarterly meetings during which the performance of the different districts and facilities are being discussed and solutions are discussed.<br>- Support supervision to the facilities from the district is being incentivized.<br>- Facilities are obliged to lower the user fees below a maximum. Patients should receive all care, lab tests and medication for this fee. It thus entails a shift from fee-for-service to case-based payment.

Abbreviations: PBF: performance-based financing; PNFP: private not-for-profit; NGO: non-governmental organization; UGX: Ugandan Shillings; ART: anti-retroviral therapy; MoH: Ministry of Health; HMIS: health management information system; HC: health center; DHMT: district health management team; MoFPED: Ministry of Finance, Planning and Economic Development; BTC: Belgian technical Cooperation; HUMC: health unit management committee; UCMB: Ugandan Catholic Medical Bureau.
